# Scoring systems for the triage and assessment of short-term cardiovascular risk in patients with acute chest pain

**DOI:** 10.31083/j.rcm2204144

**Published:** 2021-12-22

**Authors:** Nicklaus P. Ashburn, James C. O’Neill, Jason P. Stopyra, Simon A. Mahler

**Affiliations:** 1 Department of Emergency Medicine, Wake Forest School of Medicine, Medical Center Boulevard, Winston-Salem, NC 27157, USA; 2 Department of Emergency Medicine, Department of Implementation Science, Department of Epidemiology and Prevention, Wake Forest School of Medicine, Medical Center Boulevard, Winston-Salem, NC 27157, USA

**Keywords:** Risk stratification, Acute coronary syndrome (ACS), Accelerated diagnostic protocol, Risk scores, Chest pain

## Abstract

Acute chest pain is a common emergency department (ED) chief complaint. Evaluating patients for acute coronary syndrome is challenging because missing the diagnosis carries substantial morbidity, mortality, and medicolegal consequences. However, over-testing is associated with increased cost, overcrowding, and possible iatrogenic harm. Over the past two decades, multiple risk scoring systems have been developed to help emergency providers evaluate patients with acute chest pain. The ideal risk score balances safety by achieving high sensitivity and negative predictive value for major adverse cardiovascular events while also being effective in identifying a large proportion of patients for early discharge from the ED. This review examines contemporary risk scores used to risk stratify patients with acute chest pain.

## 1. Introduction

Each year 8–10 million patients visit an emergency department (ED) in the United States (US) due to acute chest pain [1, 2]. Evaluating patients with acute chest pain is a high-stakes and time-consuming process, with an estimated $10–13 billion spent annually on these evaluations [3]. Ultimately, less than 10% of ED patients with acute chest pain have acute coronary syndrome (ACS) [4–6].

Emergency department patients with acute chest pain present the emergency provider with a challenging diagnostic dilemma, especially when the majority consider a miss-rate of less than 1% acceptable, with many considering the actual threshold less than 0.5% [7]. This contributes to over-testing, which exposes patients to unnecessary, costly, and invasive procedures with possible iatrogenic harm [8–14]. Conversely, under-testing exposes the patient to potential harm from a missed diagnosis and the provider to medicolegal action. Missed ACS is a top cause of litigation against emergency providers, with payouts typically in excess of $100,000 [15, 16]. Previous ED studies suggest that emergency providers miss 2–4% of patients with ACS [17, 18]. These patients have nearly twice the mortality rate of patients with ACS who are admitted [18].

Ruling-out ACS among ED patients with acute chest pain is challenging because patient presentations vary considerably and the common diagnostic tools, such as the electrocardiogram (ECG) and troponin, are imperfect. Less than 30% of patients with ACS report the “typical” ACS symptoms, such as left sided pain, vomiting, and diaphoresis [19, 20]. Only 60% of patients with ACS describe their pain as “pressure” and only about half say their pain is worse with exertion [20]. Furthermore, the ECG is normal in up to 25% of patients with ACS [20]. Contemporary troponin (cTn) itself is also insufficient for safely ruling-out ACS, with some studies suggesting that it fails to identify nearly 10% of patients who experience a 30-day major adverse cardiovascular event [MACE; all-cause mortality, myocardial infarction (MI), or unplanned revascularization] [5, 21, 22].

Although providers may incorporate signs, symptoms, ECG findings, and troponin into their gestalt assessment, multiple prior studies describe the inadequacy of provider gestalt [22–26]. In a prospective study of provider gestalt among 458 patients presenting with chest pain, 5.6% of patients described as “Not ACS” or “Probably Not ACS” experienced a 30-day MACE [24]. Even when providers thought that a patient was “Definitely ACS”, they were right only 50.9% of the time [24]. Oliver *et al*. [25] evaluated provider gestalt at 18 sites with nearly 1400 patients. They found that gestalt for ACS, including the “Definitely Not ACS” and “Probably Not ACS” cohorts, had a sensitivity of 87.8% (95% CI 82.9–91.8) and a negative predictive value (NPV) of 94.8% (95% CI 92.8–96.3) [25]. Even when an ECG and troponin were added to gestalt, the sensitivity for detecting ACS remained unacceptably low at 86.2% (95% CI 68.4–96.1) [25].

Given the poor performance of gestalt, emergency providers have continued to pursue objective tools to help them evaluate patients with acute chest pain. Risk stratification tools aim to safely identify a large proportion of patients as low-risk and enable discharge from the ED with minimal testing. This review will focus on risk stratification methods that were derived and validated in an undifferentiated population of ED patients with acute chest pain. It will not discuss obsolete risk scoring systems that were designed to estimate risk among patients with known ACS, such as the Thrombolysis In Myocardial Infarction (TIMI) and the Global Registry of Acute Coronary Events (GRACE) risk scores [27–29].

**Fig. 1.**
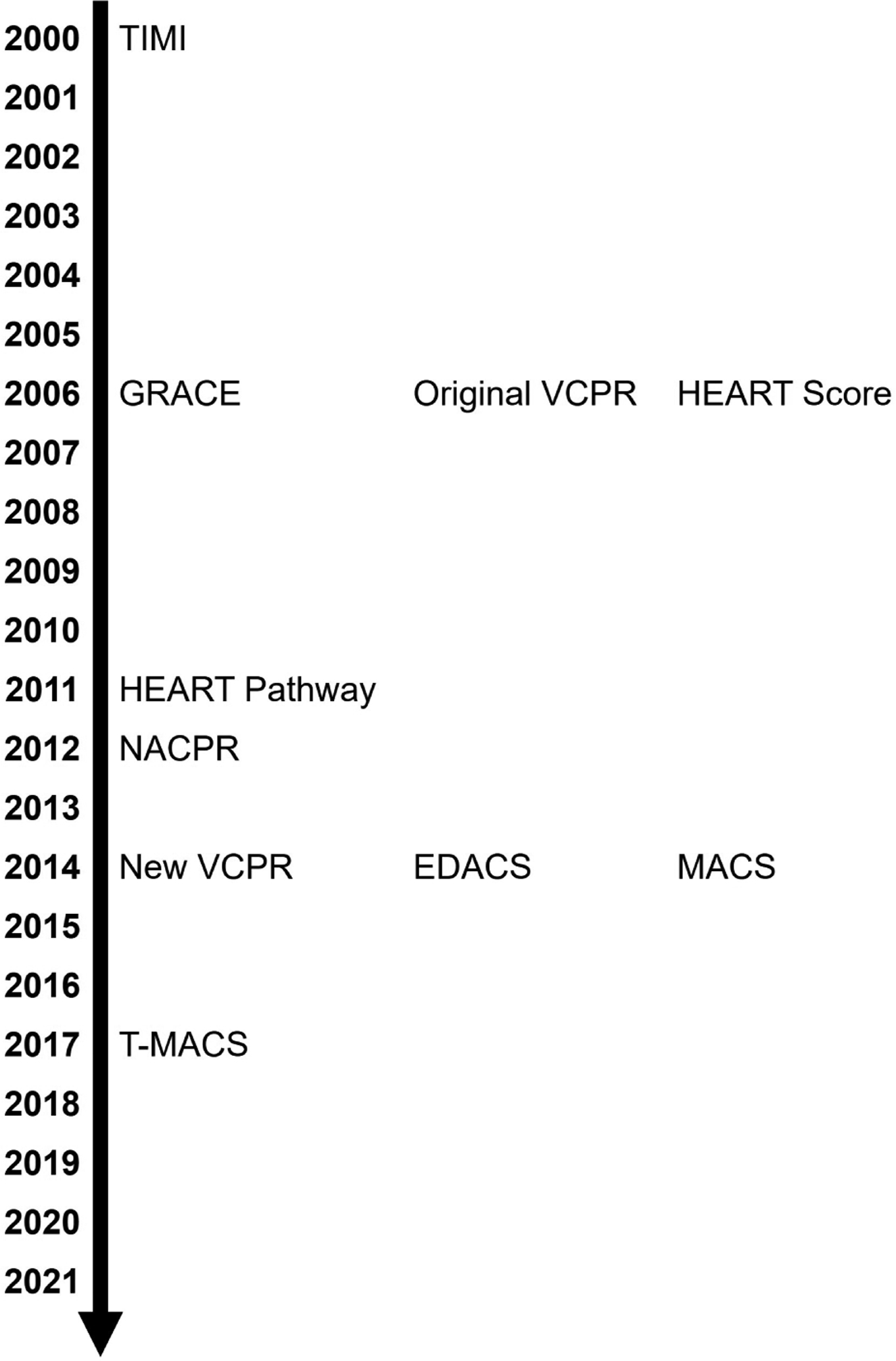
Various risk scoring systems, ordered by year of original publication.

This review will examine the Vancouver Chest Pain Rules (VCPR); the History, Electrocardiogram, Age, Risk factor, and Troponin (HEART) Score; the HEART Pathway; the North American Chest Pain Rule (NACPR); the Emergency Department Assessment of Chest Pain Score (EDACS) tools; the Manchester Acute Coronary Syndromes (MACS); and the Troponin-only Manchester Acute Coronary Syndromes (T-MACS) decision aids. Lastly, this review will discuss whether risk scores are still needed in today’s era of high-sensitivity troponin (hs-cTn) driven troponin-only algorithms. Fig. 1 shows the development timeline for these risk scores.

**Fig. 2.**
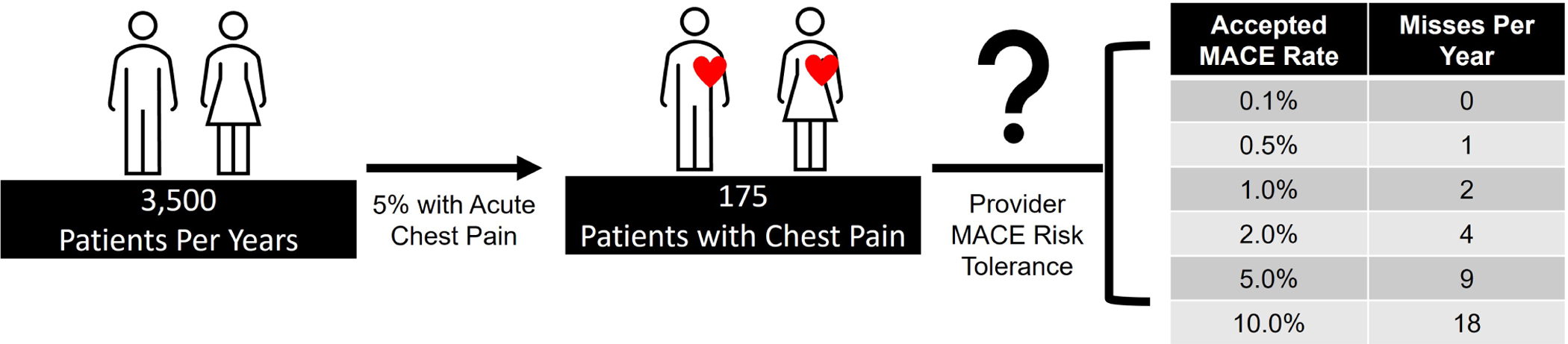
MACE events per year based on an emergency medicine provider’s risk tolerance profile.

As various risk scores and algorithms are considered, emergency providers must contemplate their own risk tolerance as well as the standard of care for their practice location. Consider the typical US emergency medicine physician who sees 3500 patients per year, of whom approximately 5% present with acute chest pain [1, 30]. Fig. 2 portrays the number of misses per year based on a provider’s accepted risk tolerance profile.

## 2. Scoring systems

### 2.1 Vancouver Chest Pain Rules

**Fig. 3.**
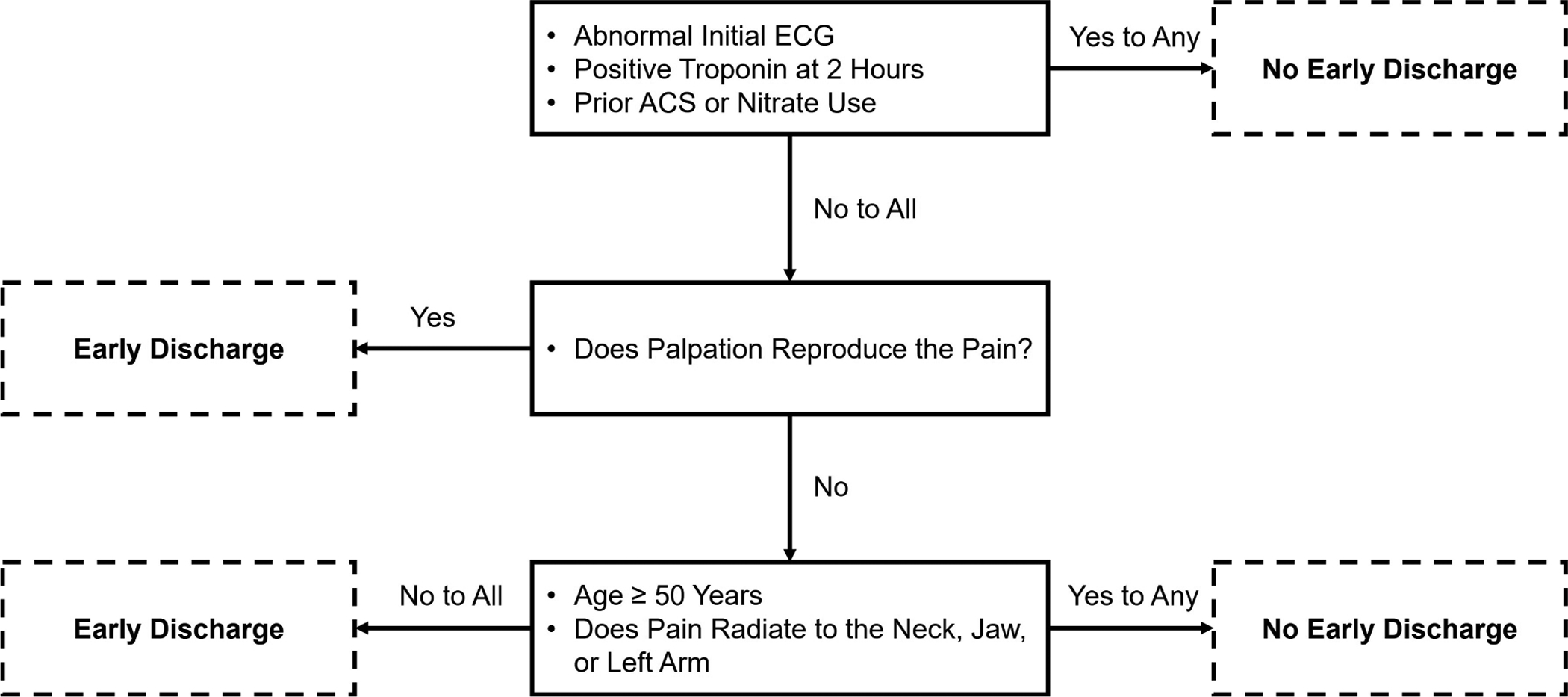
The new Vancouver Chest Pain Rule.

The original Vancouver Chest Pain Rule (VCPR) was derived from 769 patients who presented to Canadian EDs between 2000 and 2003 due to chest pain [31]. In this study, 123 variables were screened to derive a tool that identified 32.5% of patients for ED discharge with a sensitivity of 98.8% for 30-day ACS. However, this original tool relied on creatine kinase-MB (CK-MB) as the sole biomarker. Given marked advances in cardiac biomarkers, particularly the rise of troponin (cTn), an updated tool was developed. This new VCPR was derived using 53 predictor variables and incorporates cTn instead of CK-MB (Fig. 3) [32]. In the validation cohort, it identified 23.4% of patients for early discharge and was 99.2% (95% CI 95.4–100.0) sensitive for 30-day ACS. Before implementing the new VCPR, providers should consider that the sample size was not achieved for either the derivation or validation studies, it occurred in a Canadian population with increased risk tolerance compared to the US [7], the primary endpoint was 30-day ACS rather than MACE, and that the new Vancouver Chest Pain Rule was derived from data over a decade old.

### 2.2 HEART score

**Fig. 4.**
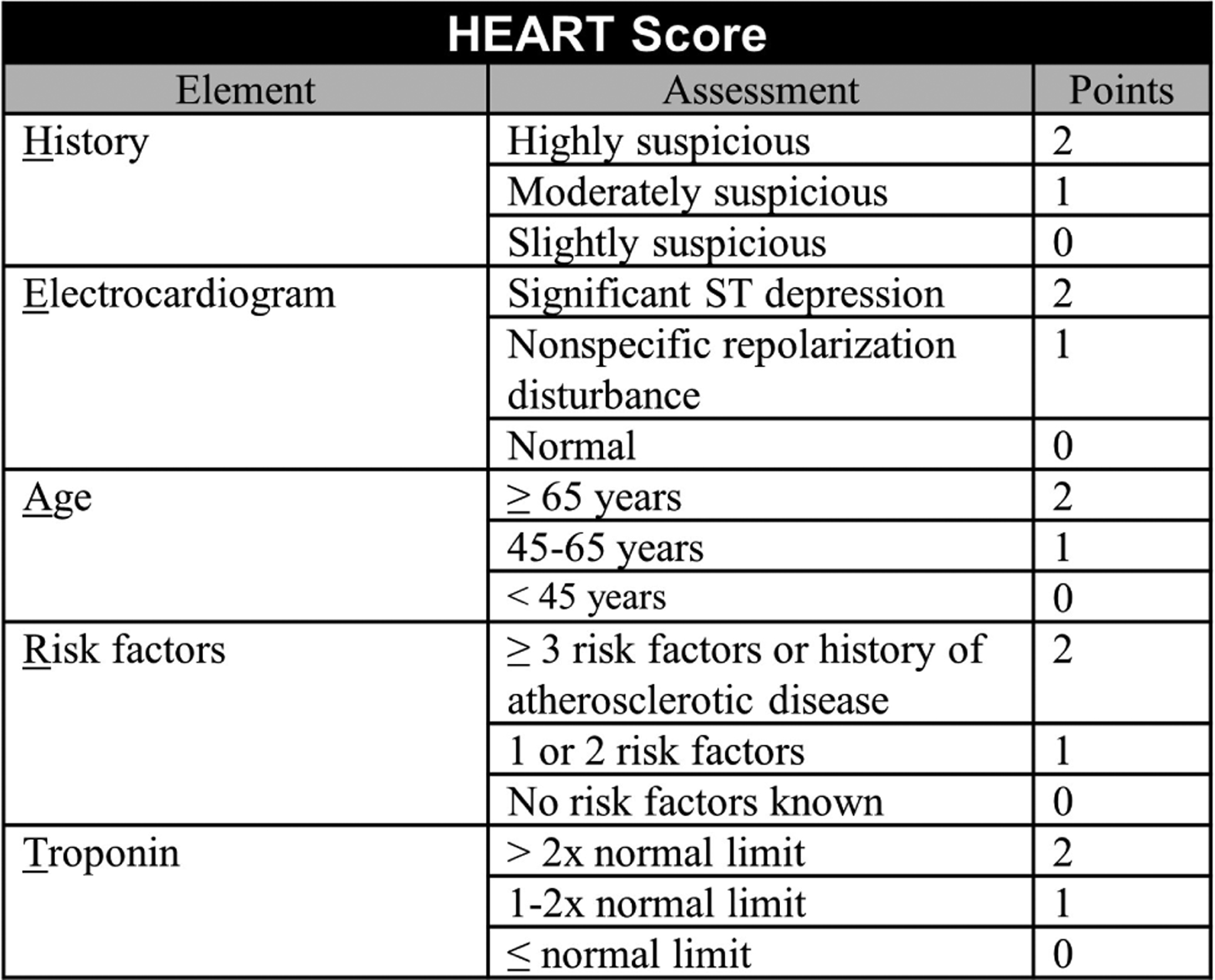
The HEART score.

In 2006, Six *et al*. [33] aimed to create an easy-to-use risk score that providers could remember and calculate without a calculator. They decided on the mnemonic HEART, with each letter corresponding to a key piece of the evaluation for patients with chest pain: History, ECG, Age, Risk factors, and Troponin. Each component was scored on a scale of 0–2, with total scores ranging between 0–10 (Fig. 4). No formal statistical derivation was used. Initially, the criteria were prospectively applied to ED patients at a single hospital in the Netherlands. The primary safety endpoint was MACE at three months. Among the 122 patients enrolled, 32.0% were classified as low-risk (HEART score 0–3). Within this group, one patient experienced a safety endpoint, suggesting that the risk of MACE was approximately 2.6%. Two years later, the HEART score was prospectively and externally validated at 10 hospitals in the Netherlands with 2388 patients [34]. Among these, 36.4% were classified as low-risk. At six weeks, 1.7% of low-risk patients experienced MACE. In 2013, nine hospitals in the Netherlands began participating in a prospective, stepped-wedge, cluster randomized trial examining the care of ED patients with acute chest pain. Of the 3648 patients in the analysis, 1827 received usual care and 1821 received care directed by the HEART score. At six weeks, 2.0% of low-risk HEART score patients experienced MACE, 1.3% fewer than the usual care group. There was no significant difference in healthcare system costs [35]. In this cohort the HEART score had a better safety profile and identified more patients for early ED discharge than two older cardiac risk scores (GRACE and TIMI scores) [36]. Multiple other studies have also demonstrated the superiority of the HEART score to GRACE and TIMI [34, 36, 37].

In 2011 the first US HEART score validation study was published. This study included patients evaluated in an ED observation unit and demonstrated that the HEART score had low sensitivity for 30-day MACE at 58.3% (95% CI 32–81). However, with the addition of a repeat troponin at 3 hours, sensitivity improved to 100% (95% CI 72–100) [38]. A secondary analysis of the prospective multicenter Myeloperoxidase In the Diagnosis of Acute coronary syndromes Study (MIDAS) also demonstrated the value in serial troponins by finding that a low-risk HEART score with serial non-elevated troponins identified 20.2% of patients for early discharge with a sensitivity of 99.1% (95% CI 96.5–100) for 30-day MACE [39]. Together these finding led to the development of the HEART Pathway, which is described separately below.

Three large HEART score meta-analyses exist. Each meta-analysis contains studies using cTn and hs-cTn. The largest examined 30 studies and 44,202 patients. It found the HEART score to have a sensitivity of 95.9% (95% CI 93.3–97.5) for MACE [40]. It also demonstrated that the HEART score had better safety and effectiveness profiles compared to the TIMI score. Another large meta-analysis of 25 HEART score studies with 25,266 patients demonstrated that the HEART score was able to classify 39.3% of patients as low-risk with a pooled sensitivity and NPV for MACE of 96% (95% CI 93–98) and 99% (95% CI 98–99), respectively [41]. A smaller meta-analysis of nine studies with 11,217 patients found a low-risk HEART score to have a sensitivity of 96.7% (95% CI 94.0–98.2) for MACE events up to six-weeks post index encounter [42].

Several limitations of the HEART score exist. First, it relies on a single troponin instead of serial troponins, making it possible to miss patients who present early with an acute MI. Based off the scoring system, it is conceivable that a patient with an elevated troponin could be classified low-risk as an elevated troponin only confers two points. Likewise, patients with ischemic ECG findings, such as ST depression or T-wave inversions, could be low-risk as these findings give a maximum of two points. Patients with a known history of coronary artery disease (CAD) could also have a maximum of two points. However, given the diagnostic implications of an elevated troponin, an ischemic ECG, or having a patient with a history of known CAD presenting with acute chest pain, discharging these patients, even if classified low-risk, seems untenable. Finally, given the subjectivity in the score, particularly in the History and ECG sections, interrater reliability is poor [43–47]. For example, in the History section, determining “Slightly” from “Moderately” and “Highly” suspicious is entirely subjective. Similarly, given the broad and generalized ECG categories, especially with “Nonspecific repolarization disturbance,” variability between providers can be expected. These subjectivity and interrater reliability concerns contribute to misclassification bias, where a low-risk patient might be categorized as non-low-risk or a non-low-risk patient may be inappropriately classified as low-risk [48]. These limitations contribute to the original HEART score having a sensitivity and NPV below what most US ED providers consider acceptable.

### 2.3 HEART Pathway

**Fig. 5.**
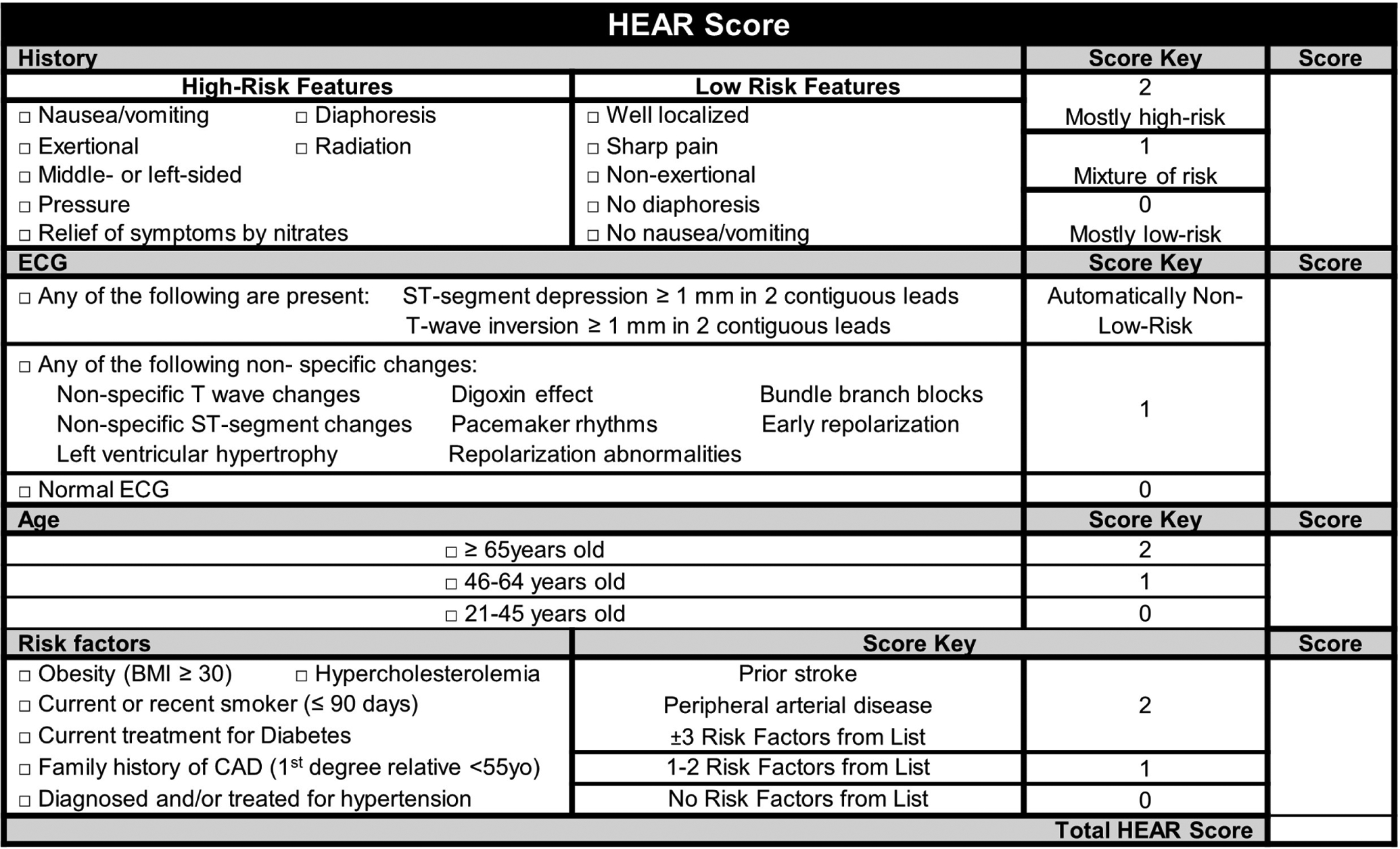
The HEAR score, a component of the HEART Pathway.

**Fig. 6.**
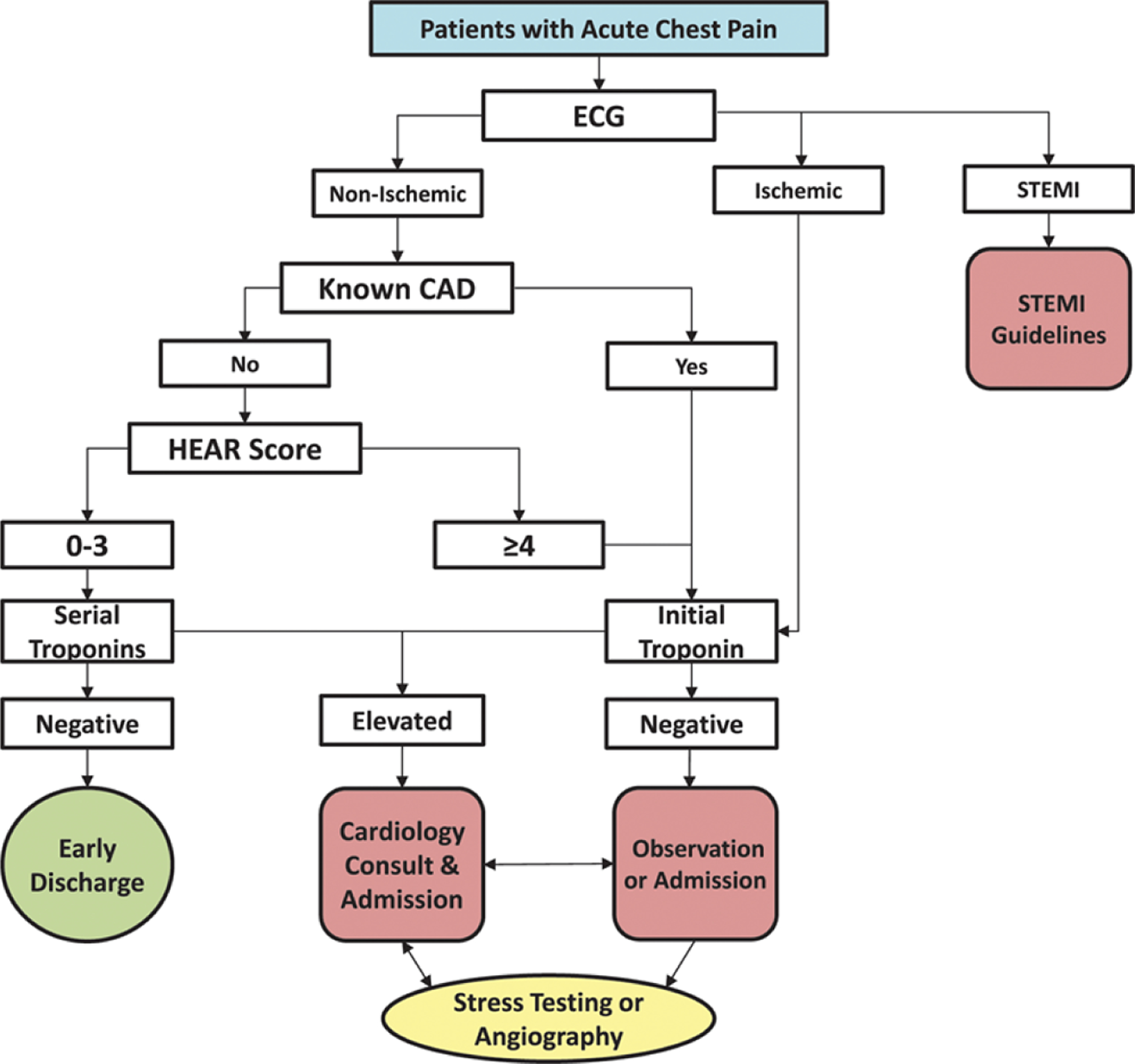
The HEART Pathway accelerated diagnostic protocol.

To improve the safety profile of the HEART score, Mahler *et al* [4, 5]. modified it by specifically addressing the limitations described above. They addressed logical inconsistencies by defining the low-risk population as patients having no known history of CAD, no ischemic ECG changes, a HEAR score of 0–3, and non-elevated serial cTns. Additionally, they made the History section an objective list of prespecified questions aimed at identifying high- vs. low-risk features, further specified concerning ECG findings, asked for explicit cardiac risk factors, and added a second cTn at 3-hours. These modifications result in a HEAR score, with results being 0–7. This HEAR score combined with serial cTn measures completes the HEART Pathway assessment. The present form of the HEART Pathway is intended to be used as part of an integrated, electronic clinical decision support (CDS) tool and is proprietary. Fig. 5 approximates HEAR score calculation and Fig. 6 shows the HEART Pathway accelerated diagnostic protocol (ADP), complete with disposition guidance.

In the 2015 HEART Pathway Randomized Trial, 282 patients were randomized to usual care or to the HEART Pathway [4]. Among the HEART Pathway patients, 46.8% were low-risk. Compared to usual care, the HEART Pathway cohort had decreased stress testing at 30 days, reduced ED length of stay, and had more early ED discharges. No low-risk HEART Pathway patient experienced 30-day MACE. In a follow-up study of one-year outcomes, Stopyra *et al*. [49] found that no low-risk patient experienced MACE, giving the HEART Pathway a sensitivity of 100% (95% CI 76.8–100.0) and a NPV of 100% (95% CI 94.6–100.0) for one-year MACE. A subsequent prospective pre-post multicenter study using the integrated HEART Pathway CDS tool accrued 8474 US patients. Of these, 30.7% were identified as low-risk, with only 0.5% experiencing 30-day MACE. This resulted in a sensitivity of 98.3% (95% CI 96.3–99.4) and a NPV of 99.6% (95% CI 99.1–99.9) [5]. An external validation study using a prospective interrupted-time-series method across 13 community hospitals in the Kaiser Permanente Southern California health system examined outcomes among 67,953 patients after implementing a HEART Pathway-like assessment. Among the post-implementation cohort, 23.6% were identified as low-risk. Of these, only 0.2% experienced 30-day death or MI. There was a 3.7% (95% CI 2.9–4.4) reduction in admission and stress testing compared to usual care [50]. An additional external prospective trial of patients with chest pain in the Henry Ford Health System evaluated another HEART Pathway-like assessment compared to usual care with observation and stress testing. At 30 days, no low-risk patient experienced an adverse event. Additionally, the patients randomized to the HEART Pathway arm had significantly less time in the hospital and fewer hospital-associated costs [51]. In an economic analysis, patients who received HEART Pathway directed care had significantly reduced costs at 30 days compared to usual care (a median reduction of $216 per individual; $1307 vs. $1523) [52]. In subgroup analyses, the HEART Pathway mitigated previously established sex and race cardiovascular care disparities, particularly among female and non-white groups [53–55]. The HEART Pathway was equally safe and effective among males and females as well as among white and non-white patients despite identifying more females and non-white patients as low-risk [56].

In summary, the HEART Pathway safely and effectively risk stratifies ED patients with acute chest pain. It overcomes the limitations associated with the HEART score, particularly undue subjectivity and logical inconsistencies such as patients with an elevated troponin, a history of CAD, or ischemic ECG findings being considered low-risk. Furthermore, the HEART Pathway has been proven to have an excellent safety profile both in internal and external validation studies among US-based cohorts. One disadvantage of the HEART Pathway is that the computer-based algorithm is proprietary. Additionally, in today’s era of hs-cTn, it is critical for providers to know that the HEART Pathway was originally validated using cTn assays. Despite this, subsequent analyses indicate similar performance when using hs-cTn [57]. Prospective implementation studies examining the HEART Pathway with serial hs-cTn are ongoing.

### 2.4 The North American Chest Pain Rule

**Fig. 7.**
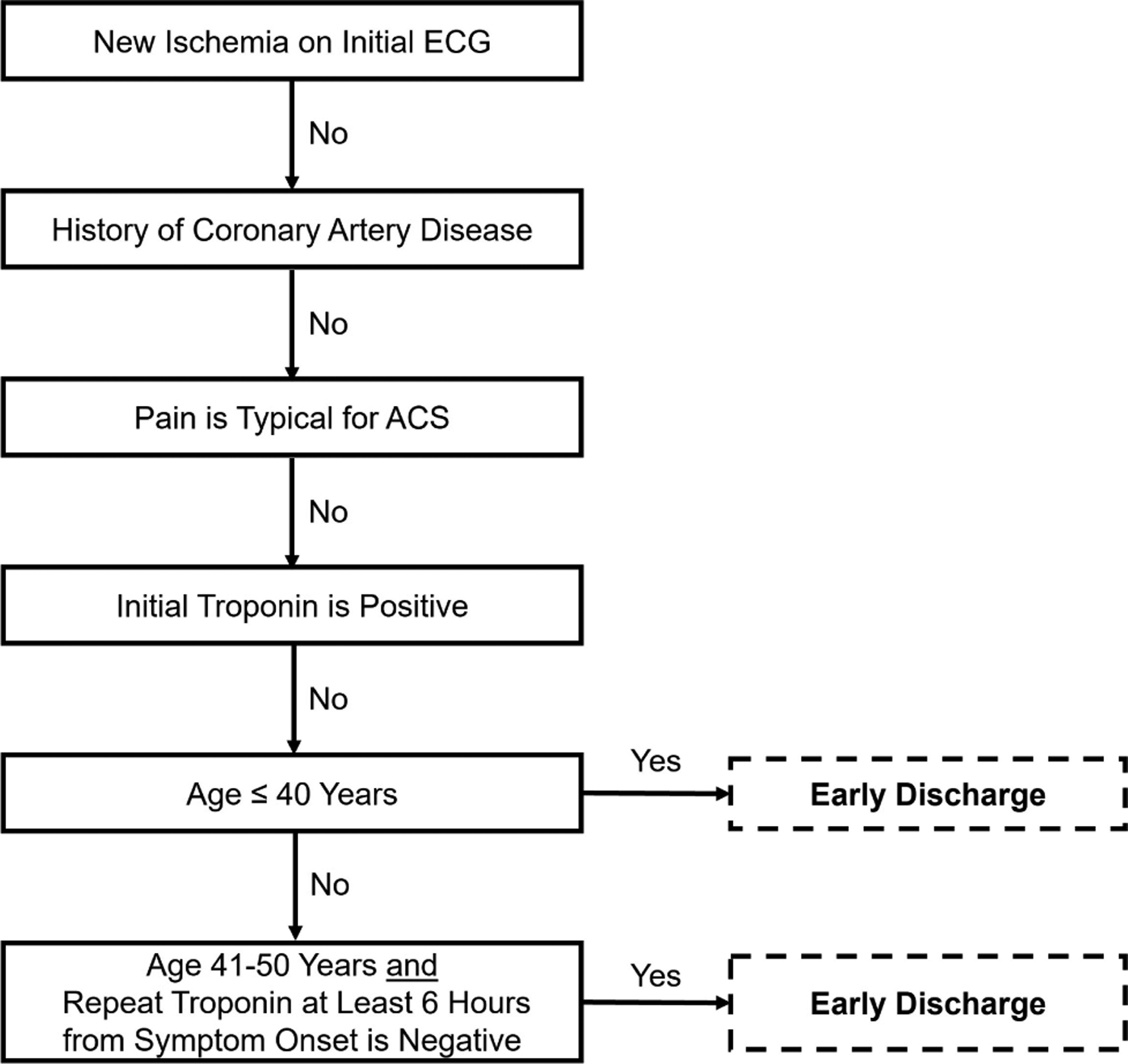
The North American Chest Pain Rule.

Hess *et al*. [20] enrolled 2718 ED patients with chest pain across two Canadian and one US site in a prospective observational study. Among these patients, 64 discrete variables were studied and eventually used to form an algorithm using recursive partitioning with the outcome of 30-day MACE. The resultant five variable algorithm is in Fig. 7. Among the 497 low-risk patients, none experienced 30-day MACE, yielding a sensitivity of 100.0% (95% CI 97.2–100.0) and a NPV of 100.0% (95% CI 99.0–100.0). In a secondary analysis of the MIDAS study, the North American Chest Pain Rule maintained 100% (95% CI 98–100) sensitivity for ACS but identified only 4% of patients for early discharge [39]. This decision tool has not been externally validated prospectively. Due to the lack of external validation, low early ED discharge percentage, and many patients requiring a 6-hour cTn measure, this decision tool is not recommended for modern-day US emergency care.

### 2.5 EDACS and EDACS-ADP

**Fig. 8.**
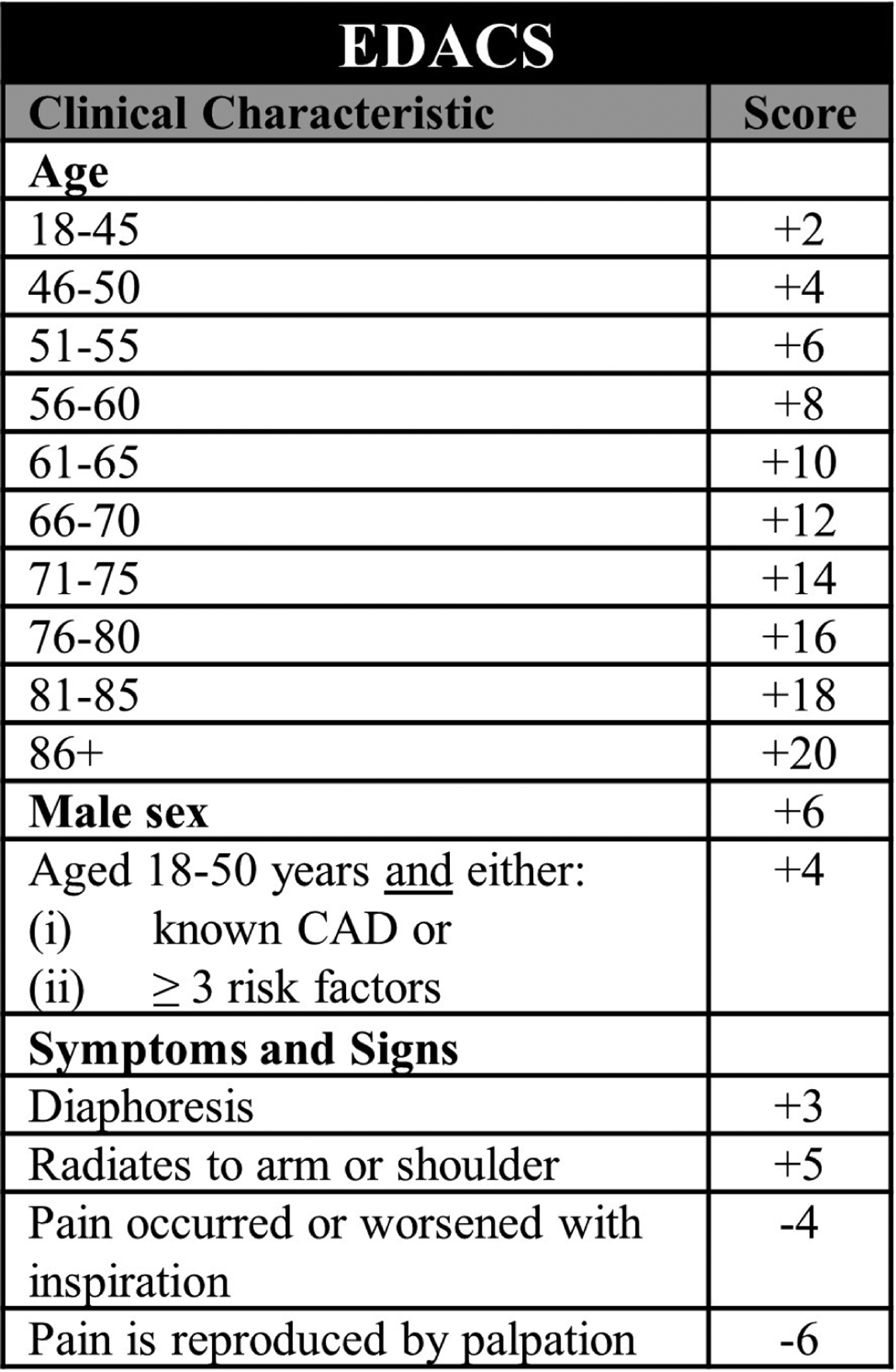
The EDACS risk score.

Than *et al*. [58] prospectively enrolled 1974 ED patients in New Zealand into a trial where 37 candidate variables as well as ECG and cTn measures were used to statistically derive both a risk score (EDACS) and an ADP (EDACS-ADP). Fig. 8 describes the risk score calculation. To be considered low-risk in the EDACS-ADP, the total score must be <16, the ECG must have no new ischemic changes, and serial cTn measures at 0- and 2-hours must be non-elevated. Than *et al*.’s [58] original 2014 study included both derivation and validation cohorts. Within these cohorts, the EDACS-ADP identified 42.2% and 51.3% of patients as low-risk and was 99.0% (95% CI 96.9–99.7) and 100.0% (95% CI 94.2–100.0) sensitive for 30-day MACE, respectively.

A recent meta-analysis of 11,578 patients across nine EDACS studies suggests that EDACS-ADP could identify 55% of patients as low-risk and achieve a pooled sensitivity of 96.1% (95% CI 89.6–98.6) and a negative likelihood ratio of 0.06 (95% CI 0.03–0.16) [59]. Given the pretest probability of 30-day MACE in the combined cohort, the authors concluded that the posttest probability of 30-day MACE is <1% for patients identified as low-risk by the EDACS-ADP.

In a secondary analysis of 282 patients with acute chest pain in US EDs, the EDACS-ADP was 88.2% (95% CI 63.6–98.5) sensitive for 30-day MACE and had a NPV of 98.9% (95% CI 96.2–99.9) [60]. In another US ED-based study of 4399 patients, Stopyra *et al*. [61] conclude that while EDACS identified more patients as low-risk compared to the HEART Pathway [58.1% (95% CI 56.6–59.6) vs. 38.4% (95% CI 37.0–39.9)], it suffered the tradeoff of increased 30-day MACE [1.0%, (95% CI 0.7–1.5) vs. 0.4% (95% CI 0.2–0.9)]. Although statistically derived and relatively easy to calculate, US providers must acknowledge that the tool was originally designed in and seems to perform optimally in Australasian settings. Additionally, no interrater reliability data is available. Given these limitations, particularly its mixed safety profile among US patients, emergency providers may desire additional prospective US data prior to incorporating EDACS into their daily practice.

### 2.6 MACS and T-MACS

**Fig. 9.**
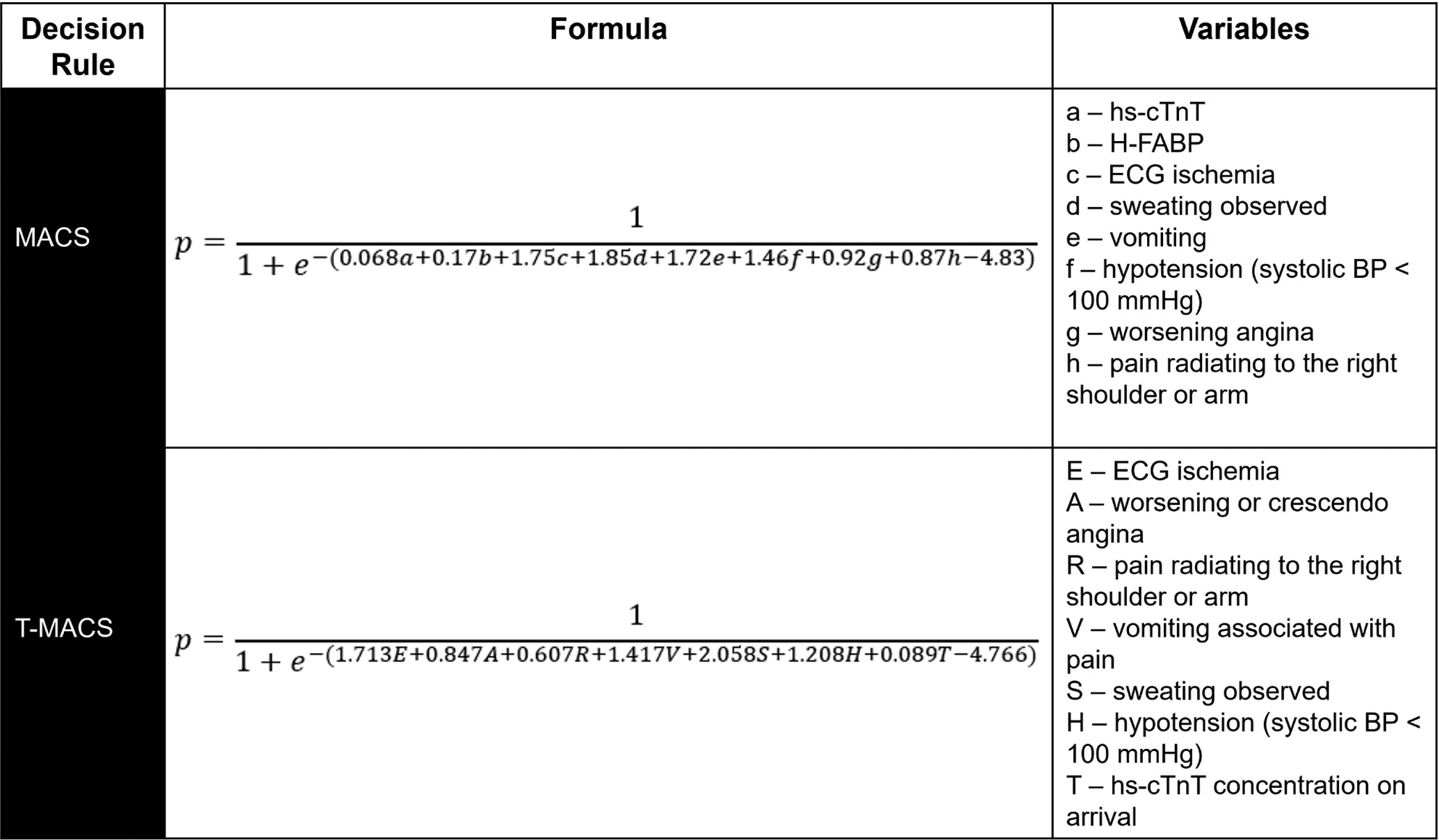
Probability (p) of ACS per MACS and T-MACS. Variables are binary (yes = 1, no = 0) except for hs-cTnT and H-FABP concentrations and age. Variable names, order, and descriptions are per the original derivation papers.

The Manchester Acute Coronary Syndromes (MACS) clinical decision rule was derived using logistic regression in a study of 698 English ED patients with acute chest pain [62]. Eight key variables were identified, including two biomarkers: high-sensitivity troponin T (hs-cTnT) and heart-type fatty acid binding protein (H-FABP). Patients were stratified into four groups: very low-risk (suitable for ED discharge), low-risk (ED or ED observation unit with serial hs-cTnT), moderate risk (admission with serial hs-cTnT), and high-risk (ACS ruled-in). In the original derivation study, 35.5% of patient were “very low-risk”, suggesting that they could be discharged directly from the ED (see full model in Fig. 9). When used in binary fashion for deciding to discharge or admit patients, it had a sensitivity of 99.4% (95% CI 96.5–100.0) and a NPV of 99.6% (95% CI 97.8–100.0) for 30-day MACE.

Given the limited availability of H-FABP, Body *et al*. [63] performed secondary analyses on four prospective datasets to re-derive and re-validate a version of MACS without H-FABP, with the new tool termed the Troponin-only Manchester Acute Coronary Syndromes (T-MACS) decision aid. It relies on seven key variables, including hs-cTnT. T-MACS estimates the probability of ACS using the formula in Fig. 9. The same four risk categories from MACS apply, with very low-risk defined as *p* < 0.02, low-risk 0.02 < *p* < 0.05, moderate-risk 0.05 < *p* < 0.95, and high-risk as *p* ≥ 0.95 or with ACS ruled-in. In the re-derivation set, 37.7% of patients were very low-risk and suitable for ED discharge. The sensitivity and NPV for 30-day MACE among very low-risk patients were 98.7 (95% CI 95.3–99.8) and 99.3 (95% CI 97.3–99.9). In an external validation study using prospectively collected data among ED patients in Australia and New Zealand, T-MACS identified 19.8% of patients as very low-risk and was 96.3% (95% CI 92.2–98.6) sensitive and had a NPV of 97.6% (95% CI 94.8–99.1) for 30-day MACE [64]. With nearly 40% of patients identified as suitable for ED discharge in the original study, a miss rate approaching 1%, and owing to the tool’s ease-of-use, T-MACS may be a reasonable risk stratification tool. However, no validation study among a US-based cohort presently exists. Given this, more data are needed before implementing T-MACS into routine practice in the US.

### 2.7 Test characteristics

The test characteristics of each risk score and ADP described above are summarized in Table 1 (Ref. [4, 5, 20, 32, 33, 37–40, 42, 60–68]).

## 3. The (uncertain) future of risk scores in the era of high-sensitivity troponin

Recently, widespread implementation of high sensitivity cardiac troponin assays (hs-cTn) has increased interest in the use of “troponin-only” ADPs, which do not incorporate a formal risk score. Protocols like the European Society of Cardiology’s (ESC) 0/1-hour algorithm and the High-STEACS Pathway classify patients into three zones: rule-out, observe, or rule-in based solely on the initial and serial hs-cTn results. These algorithms boast of being able to classify nearly 50% of patients as low-risk and as acceptable for early discharge with very few misses [69–75]. However, there is little data on the performance of hs-cTn only algorithms in US ED settings. Furthermore, data evaluating whether risk scores add value to these algorithms is conflicting.

The HIGH-US trial was the first to validate the ESC 0/1-hour algorithm in US EDs and demonstrated high sensitivity for 30-day death and MI [73]. However, in the STOP-CP trial the hs-cTnT ESC 0/1-hour algorithm was unable to achieve acceptable sensitivity and NPV for 30-day MACE, which suggests that it may not be safe for use in the US [76]. Importantly, when the ESC 0/1-hour algorithm was combined with a low-risk HEART score, sensitivity and NPV improved significantly, suggesting a remaining important role for risk scores in the era of hs-cTn [76]. Similarly, Mokhtari *et al*. [77] found the ESC 0/1-hour algorithm to have a low sensitivity (87.6%, 95% CI 80.4–92.9) for 30-day MACE, which improved (97.5%, 95% CI 92.9–99.5) with the addition an ECG and clinician risk assessment. However, other studies have reported no significant improvement in the diagnostic performance of hs-cTn only algorithms when adding a risk score [78, 79]. While, the value of risk scores in the era of hs-cTn is the subject of ongoing debate, these mixed safety results should give US providers pause before adopting a hs-cTn only chest pain risk stratification algorithm [80–82].

## 4. Conclusions

When caring for patients with acute chest pain, emergency providers have an array of risk stratification tools at their disposal. Each tool has unique benefits as well as key limitations. Local standards of care and provider risk tolerance should determine which tool is used. However, there is no perfect tool, thus risk scores and ADPs are not meant to eliminate provider judgment. Although novel hs-cTn only algorithms may identify more patients for early discharge, US-based prospective safety data are mixed, suggesting that relying solely on these algorithms may expose patients and providers to unnecessary and avoidable risks. The highest quality evidence indicates that the safest care is provided by combining risk scores with serial cTn measurements. The HEART Pathway has been proven safe and effective among US patients both in internal and external validation studies conducted across multiple sites, institutions, and among thousands of patients. Even so, given that the field of chest pain risk stratification research is rapidly maturing, safer and more efficient approaches are likely on the horizon.

## Figures and Tables

**Table 1.** Test characteristic ranges of each risk stratification tool for 30-day MACE or ACS.

Risk stratification method	Sensitivity	Specificity	PPV	NPV
New Vancouver Chest Pain Rule [32, 65, 66]	98.6–100%	16.1–37.3%	7.4–23.3%	98.6–100%
HEART score [37, 38, 40, 42, 67]	58.3–96.6%	31.8–85.0%	4.2–41.2%	96.6–99.4%
HEART pathway [4, 5, 61, 65]	95.0–100%	39.9–55.9%	10.3–16.3%	99.6–100%
North American Chest Pain Rule [20, 39]	100%	5.6–20.9%	15.1–23.2%	100%
EDACS-ADP [32, 33, 60, 61, 65]	86.3–100%	49.9–70.2%	13.8–26.5%	98.9–99.8%
MACS [62–64]	98.2–99.4%	12.0–47.6%	14.4–34.7%	97.7–99.6%
T-MACS [63, 64, 68]	95.2–98.7%	22.7–47.6%	15.7–34.0%	97.4–99.3%

PPV, positive predictive value; NPV, negative predictive value.
